# “Calculating faces”: can face perception paradigms enrich dyscalculia research?

**DOI:** 10.3389/fpsyg.2023.1218124

**Published:** 2024-01-03

**Authors:** Maria Baulina, Vladimir Kosonogov

**Affiliations:** ^1^Laboratory of Counseling Psychology and Psychotherapy, Federal Scientific Center of Psychological and Multidisciplinary Research, Moscow, Russia; ^2^International Laboratory of Social Neurobiology, HSE University, Moscow, Russia

**Keywords:** dyscalculia, facial recognition, learning disability, visual perception, brain

## Abstract

Developmental dyscalculia (DD) is a subtype of learning disabilities, which is characterized by lower mathematical skills despite average intelligence and average or satisfactory performance in other academic areas. It is not fully understood how such deficits emerge in the course of brain development. When considering the mechanisms of dyscalculia, two domain-specific systems are distinguished. The Approximate Number System (ANS) is related to the approximate estimation of large sets, and the Object Tracking System (OTS) is responsible for subitizing, that is, the exact quantification of small sets. In recent years, the multiple-deficit framework has become increasingly popular. On the one hand, it explains the impairment of certain general cognitive functions in children with DD, such as executive functions, attention, visual-perceptual discrimination, processing speed, and rapid scanning of visual information. On the other hand, it provides a theoretical basis for explaining the simultaneous occurrence of the different types of other comorbid conditions (such as dyslexia and ADHD) and the relationship between them. We suggest that the face recognition could be considered as another, probably impaired function in dyscalculic individuals. We highlight several brain areas involved both in numerical and facial processing: intraparietal sulcus (IPS), fusiform gyrus (FFG), and hippocampus (HC). We consider the possibility of expanding the scope of dyscalculia research by application of face perception paradigms.

## Introduction

Developmental dyscalculia (DD) is a subtype of learning disabilities characterized by lower mathematical skills. Its prevalence ranges between 3 and 8% of the population (Geary, 2004; Rubinsten and Henik, 2009; Rapin, 2016; Skagerlund and Träff, 2016). Mathematical performance depends on the specific numerical skills required in each area of mathematics and general cognitive abilities, such as symbolic number processing and visuospatial working memory. Many studies support the idea of domain-specific deficit in dyscalculic learners due to the difficulties in understanding basic numerical concepts, such as magnitude comparison or accounting. Generally speaking, there are two systems of quantity estimation according to the domain-specific approach to dyscalculia. The first system, Approximate Number System (ANS) supports the approximate estimation of large sets (Halberda and Feigenson, 2008; Bugden and Ansari, 2016). The second one, Object Tracking System (OTS), is supposed to be responsible for the exact quantification of small sets (subitizing) (Fischer et al., 2008; Henik et al., 2011).

The etiology of developmental disorders can also be considered within the multiple-deficit framework (Pennington, 2006; Moll et al., 2019; McGrath et al., 2020). It provides a theoretical basis explaining the simultaneous occurrence of disorders and the relationship between them. As for dyscalculia, this approach takes into account other comorbid conditions. Children with dyscalculia also have attention deficit hyperactivity disorder (ADHD) or dyslexia in 5–30% (DuPaul et al., 2012; Georgitsi et al., 2021), and in 11–70% of dyscalculic learners, respectively (Moll et al., 2014; Wilson et al., 2015; Peters et al., 2020). Some functions are also impaired in children with DD, for example, executive functions, visual-perceptual discrimination, processing speed and rapid scanning of visual information (Geary, 2011; Child et al., 2019). It is possible to distinguish children with specific deficits in mathematics and the children for whom difficulties with mathematics are the result of problems in other cognitive domains. In the first case, rehabilitation could directly address mathematical skills, whereas rehabilitation could be aimed at the impaired general cognitive function. The comorbidity between dyslexia and dyscalculia deserves special attention (Mann Koepke and Miller, 2013; Moll et al., 2021), since dyslexia is the closest impairment to dyscalculia. They have a possible common genetic precursor, the deletion of the chromosome 15q11.2, which is associated with high risk of dyslexia or dyscalculia (Ulfarsson et al., 2017).

Little research has directly compared the cognitive abilities in dyscalculia and dyslexia. The studies targeted the investigation of symbolic and non-symbolic numerical magnitude, phonological awareness, attention, verbal short-term memory, visuospatial memory, lexical access, and working memory (Geary et al., 2020; Grant et al., 2020). Peters et al. (2020) demonstrated that children with dyscalculia had a marked deterioration in spatial skills in comparison to their typically developing peers. Here, spatial skills were a strong predictor of isolated dyscalculia and comorbid dyslexia/dyscalculia, while phonological awareness was the only reliable indicator of isolated dyslexia. Neural activation patterns in children with comorbid dyslexia/dyscalculia and isolated dyslexia and dyscalculia, obtained with fMRI, had similar differences in brain activity compared to their typically developing peers during arithmetic tasks (Georgitsi et al., 2021).

### Insights from neuroscience: brain regions critical for dyscalculia

Research shows that several brain regions are critical for dyscalculia. This article focuses on three areas. First, many researchers suggest that the parietal cortex, especially the intraparietal sulcus (IPS), plays a critical role in dyscalculia. von Aster and Shalev (2007) assumed that functional specialization of the parietal cortex in mental calculations enhanced with age and was combined with a reduction in prefrontal activity. This supposition coincides with the experience-dependent neuroplastic effect on the IPS in children with typical ontogenesis. Henik et al. (2011) noted the role of IPS in dyscalculia, but also believed that DD could be characterized by other impairments, has heterogeneous symptoms, and could be influenced by domain-independent factors related to other brain areas. Michels et al. (2021) compared whole-brain maps of volume based structural covariance between control and dyscalculic groups and detected high structural covariance in children with DD between the anterior IPS and the middle temporal and frontal gyri. They found a bilateral involvement of the IPS, with significant deterioration of the left IPS, in older schoolchildren with DD. There was decreased probability of connections from the right fusiform gyrus (FFG) to the parietal lobes and other brain areas. Butterworth et al. (2011) found reduced activation of the IPS in DD group during tasks for number symbols or numerosity comparison and arithmetic. Differences in the activation between children with dyscalculia and the control group were detected in the occipital and frontal cortex along with the parietal areas associated with numbers. Rykhlevskaia et al. (2009), using structural MRI and diffusion tensor imaging to estimate structural impairments in children with DD, found reduced gray matter bilaterally in the superior parietal lobule, IPS, FFG, parahippocampal gyrus, in the hippocampus, and right anterior temporal cortex. In a meta-analysis, Kaufmann et al. (2011) demonstrated that dyscalculic individuals produced consistent activation in frontoparietal areas in response to calculating tasks and number processing that were distinctly modulated by notation, the level of competence and task complexity. Pinel and Dehaene (2013) studied, using fMRI, a group of monozygotic and dizygotic adult twins during a mental calculation task. The patterns of activation were under genetic influence, encompassing the bilateral posterior superior parietal lobules, the right IPS, and a left superior frontal region. Also, the main impact of the shared environment was found in the lateralization of activation within the IPS.

Second, a number of studies highlighted the role of the FFG in number processing. Kucian et al. (2011), in a fMRI study, displayed differences in brain activation in the supplementary motor area and the right FFG, where children with DD demonstrated stronger activation compared to the control subjects. The authors considered this result to be a developmental impairment of a spatial number representation. In a study by Vatansever et al. (2020), group differences were observed in the posterior insula, FFG, and peristriate cortex, which correspond to the number form area in ventral occipitotemporal cortex. The very existence of this area remains debatable (Merkley et al., 2019).

Third, the hippocampus (HC) is typically related to working memory and encoding complex visual stimuli during numerical tasks (Peters and De Smedt, 2018). In an fMRI study by Üstün et al. (2021), the participants performed a quantity dot and symbol comparison in two levels of complexity and children with dyscalculia demonstrated neural activation in the left hippocampus for the symbolic condition. Reverse meta-analysis of functional connectivity in fMRI studies showed the strength of pretraining interregional coactivations between IPS and HC in quantity discrimination, and associative learning predicted individual variability in number sense learning across children with math difficulties and the control group (Chang et al., 2022). Geduk et al. (2020) considered effective connectivity in dyscalculia in the prefrontal-parietal and hippocampal network in the left hemisphere as a result of their brain compensation mechanisms.

### Where dyscalculia and face perception overlap in the brain?

Recent studies have shown that these same brain regions, which we highlighted for DD, are also involved in face perception. This is partly due to the tendency to expand the brain areas involved in mathematical processes (Arsalidou and Taylor, 2011). Narumoto et al. (2001) found areas that responded more to faces than to non-face stimuli: the bilateral IPS along with regions such as the bilateral FFG and the right superior temporal sulcus. Dehaghani et al. (2022), in a magnetoencephalographic study, presented a 1-back task in which it was necessary to determine if the two sequential faces (of humans and monkeys) and non-face stimuli were the same or not. It was found that the pre-stimulus activity in the left occipital area caused the activity in the IPS after the presentation of a human face in approximately 170 ms. Bzdok et al. (2012) delineated the neural functional networks activated in 4 facial judgments: cognitive (age), social (attractiveness, trustworthiness) and emotional (happiness) features. This led to the activation of IPS along with superior parietal cortex, bilateral activation in the premotor and supplementary motor cortex, in the ventral visual cortex including the inferior occipital gyrus, the middle occipital, lingual, and fusiform gyri. Repetitive transcranial magnetic stimulation in right IPS improved facial emotion recognition in the left visual area. It could result in the amplification in the functional connectivity between intraparietal and superior temporal sulcus, and visual cortex. The stimulation created conditions for emotional stimuli to mobilize attention resources until the conscious awareness (Fan et al., 2018).

The role of the FFG in face recognition is no longer in doubt. Early neurophysiologic recordings in monkeys found face responses in the inferotemporal cortex (Perrett et al., 1992). Modern studies in humans have been carried out using intracortical EEG from epileptic patients viewing a sequence from a neutral face to fearful or happy. It showed that N200 field potential peak latencies indicated that face processing begins in the inferior occipital cortex and proceeds anteroventrally to fusiform and inferior temporal cortices in parallel (Babo-Rebelo et al., 2022). FMRI highlights the fusiform face area (FFA) that is specific to the perception of faces (Kanwisher et al., 1997; Kanwisher and Yovel, 2006). A growing number of studies reveal the connection of FFA not only with face recognition, but also with facial emotion recognition (Guyer et al., 2008; Thome et al., 2022). Zinchenko et al. (2018), in a meta-analysis of fMRI, suggested that dynamic stimuli (e.g., videos) may be more specific and ecologically valid to study face perception. They concluded that the consistency of the brain regions, often mentioned in the context of facial recognition including FFG and posterior parts of the superior temporal gyrus, areas associated with more general processes in facial processing, such as the left amygdala, the anterior parts of the superior temporal and inferior frontal gyri, as well as a part of the cerebellar declive. Both structural and functional MRI demonstrated that reduced size of the left FFG and atypical activation in the left angular and left fusiform gyri are also matched the 15q11.2 deletion, critical for both dyscalculia and dyslexia (Ulfarsson et al., 2017).

Recently, the hippocampus was considered as a brain region that makes a contribution to facial emotion recognition (Üstün et al., 2021; Kuang et al., 2022). Current data suggest that HC is involved in both visual streams: in the ventrolateral visual stream for objects and faces (“what”) and in the ventromedial visual stream for scene recognition (“where”) (Rolls et al., 2023). Fried et al. (1997) demonstrated that single НС neurons distinguished faces from inanimate objects during encoding and recognition and some units reacted selectively to conjunctions of facial expression and gender or facial expressions only. A systematic review of brain activity during the tasks which could cause anger and aggression in participants with a history of aggression, demonstrated enhanced responses in the left hippocampus and parahippocampal gyrus, and left amygdala (Nikolic et al., 2022). In an fMRI study of healthy adults in groups of different ages, while perceiving faces paired with labels for different expressions (happy, neutral, or angry), the coherence between НС and orbital frontal cortex revealed subsequent memory effects for the happy condition in both groups. An HC-FFA network was identified for neutral and happy conditions in young participants and interactions between HC and posterior superior temporal sulcus for happiness in the older group (Izumika et al., 2022).

### Face perception in dyscalculia

Studies of face perception in dyscalculia are contradictory. At the same time, systematic empirical research has not been conducted. For example, De Visscher et al. (2018) showed that categorization of four faces created by morphing two young females in DD did not differ from the control group. At the same time, dyscalculia has been mentioned in prosopagnosia research. Dalrymple et al. (2012) believed that the progress in developmental prosopagnosia research may also provide insights about other selective developmental deficits such as dyscalculia, dyslexia, and specific language impairment. Ramus (2004) proposed a theory stating that some developmental disorders, such as developmental prosopagnosia, dyslexia and dyscalculia are the consequence of such a neurobiological problem as neural migration errors. It leads to a focal cortical impairment and behavioral deficit.

Empirically speaking, in a survey of adults with prosopagnosia, 57% of them reported at least one developmental comorbidity, with most reflecting specific cognitive impairments, such as object agnosia, memory impairment, and navigation problems (Svart and Starrfelt, 2022). However, only 3.5% of the sample reported comorbid dyscalculia. At the same time, an experimental part has not been conducted by the authors.

Decarli (2019) presented numerical and face perception tasks to 12-month-old infants. The aim was to verify the longitudinal specificity of ANS as a unique predictor of early symbolic and nonsymbolic numerical achievement, and to understand whether a positive correlation could be liable to differences in discrimination of quantities or to more general perceptual abilities. Face recognition was used as a control task, since the author supported the idea of different cortical streams for these processes - ventral for faces and dorsal for numbers (Golarai et al., 2007; Chinello et al., 2013). There were no significant correlations between the results in the numerical and face perception tasks at differing levels of complexity. The author concluded the dissociation between numerical acuity and the face perception ability and supposed that they rely on separate pathways. Of note, only infants were studied, but not schoolchildren with dyscalculia.

Despite the traditional ideas about angular gyrus as part of the dorsal stream (Cooper and O’Sullivan, 2016), Rapin (2016), describing Gerstmann syndrome, noted that it was associated with the ventral visual stream, involved in the recognition of familiar objects, colors and faces. The author described supramarginal and angular gyrus, where information is transmitted about written symbols, such as letters, Arabic numerals, mathematical or chemical formulas, that the working memory extracts online from long-term storage. McIntosh and Schenk (2009) noted that the idea that these streams work largely independently of each other may not be entirely true and it is necessary to consider more closely how brain areas are integrated from task to task into new functional networks.

### Face perception in dyslexia: what can be adopted by dyscalculia research

Finally, as dyslexia is closely related to dyscalculia, it is relevant to briefly outline the findings on face perception in dyslexic individuals. Early studies described the specific features of faces that were drawn, by children with dyslexia, that are associated with spatial difficulties (Pontius, 1976, 1981). The main findings of the contemporary research suppose that persons with dyslexia have mild impairment in visual tasks including face and word perception, which lie in holistic processing (Gabay et al., 2017; Brady et al., 2021; Åsberg Johnels et al., 2022). The difficulties in reading may be considered as a more general high-level visual deficit, that, in line with dyscalculia, is related to functional impairments within the left FFG, a part of the ventral visual stream (Tarkiainen et al., 2003; Sigurdardottir et al., 2015, 2018, 2021).

Jozranjbar et al. (2021) demonstrated that it was hard for dyslexic readers to recognize objects (e.g., images of houses), pointing out that visual difficulties in dyslexia may be considered not only as domain-specific. Reduced accuracy of houses recognition was combined with reading difficulties while face recognition was not impaired, which could confirm the connection between visual word processing and visual processing of non-facial objects. Featural and configural processes coexisted in the control group, while dyslexic individuals relied on a single process. This phenomenon was observed for both faces and houses.

The literature on developmental and acquired prosopagnosia considers the models of visual perception related to more general processes in face and word recognition. The data obtained are the subject of discussion among researchers. In some studies, there is no connection between the processing of faces and words (Rubino et al., 2016; Burns et al., 2017; Kühn et al., 2021; Gerlach et al., 2022; Gerlach and Starrfelt, 2022). For example, Rubino et al. (2016) showed in their study normal visual text processing in all studied subjects with developmental prosopagnosia, except one. However, other data acquired on prosopagnosia demonstrated a significant connectivity between word and face perception: impairments in face recognition were related to poorer word processing (Burns and Bukach, 2021, 2022; Sigurdardottir et al., 2021). However, children with dyslexia exhibit general difficulties in bimodal speech perception, apparent in them lacking any benefit from bimodal information (i. e., bimodal perception of video-recorded mouths pronouncing syllables). This general deficit in children with dyslexia may underlie reduced bimodal benefit for letter-speech sound combinations in emotion perception (Creusere et al., 2004; Schaadt et al., 2019).

Some researchers associate the features of emotional expression recognition with specific eye movements, and demonstrate that lower performance in emotional face processing in children with dyslexia could be due to a difference in their visual strategies, linked to recognition of unpleasant facial expressions (Goulème et al., 2017; Liu et al., 2017). Some publications mentioned the difficulties of learning in general, comparing nonverbal and verbal learning disabilities (Dimitrovsky et al., 2000; Bloom and Heath, 2010). The necessity of studying emotion recognition in schoolchildren with different subtypes of verbal learning disabilities is noted.

## Conclusions and suggestions

Up to date, the relationship between dyscalculia and face processing has been studied inconsistently and insufficiently. Many studies support the idea of a domain-specific deficit in children with dyscalculia due to the difficulties in understanding basic numeric concepts, such as magnitude comparison or accounting. However, the etiology of developmental disorders can be considered within the multiple-deficit framework. A glance into brain underpinnings of both number and face processing allows us to emphasize areas where these processes overlap. First, it is a wide number of parietal regions, especially the IPS. Second, the FFA which was previously specifically considered to provide facial recognition, has recently been also related to number processing. Third, HC may be engaged in memory processes, required for both number and face processing. However, since particular brain region is responsible for multiple cognitive functions, impairment in one function may not be related to other. Nevertheless, it can be assumed that DD and face perception may overlap in the aspect of assessing spatial information. The preliminary surveys suggest a low frequency of dyscalculia in people with prosopagnosia. Scarce literature on the relationship between face perception and dyscalculia is either contradictory. However, systematic empirical research has not addressed this potential association. Remarkably, research on dyslexia, the most comorbid impairment, suggests the potential to broaden the scope of dyscalculia studies thanks to the application of face perception paradigms. This would presumably help us to define the extent and frequency of face perception impairments in DD. To provide a clear idea of the relationship between face perception and dyscalculia, experiments are needed that include comparing the features of all types of visuospatial perception in children with DD.

## Author contributions

MB: writing – original draft. VK: writing – review and editing. All authors contributed to the article and approved the submitted version.
